# Why the trial researcher matters: Day-to-day work viewed through the lens of normalization process theory

**DOI:** 10.1016/j.ssmqr.2023.100254

**Published:** 2023-06

**Authors:** Lindsay Dalgarno, Linda Birt, Christine Bond, Jeanette Blacklock, Annie Blyth, Jacqueline Inch, Frances Notman, Amrit Daffu-O’Reilly, Maureen Spargo, Laura Watts, David Wright, Fiona Poland

**Affiliations:** a Institute of Applied Health Sciences, University of Aberdeen, Scotland, UK; b General Practice and Primary Care, University of Glasgow, Scotland, UK; c School Health Sciences, University of East Anglia, UK; d School of Healthcare, University of Leicester, UK; e School of Pharmacy, University of East Anglia, UK; f School of Healthcare, University of Leeds, UK; g School of Pharmacy, Queen's University, Belfast, UK

**Keywords:** Pharmacists, Care homes, Research activity, Documentary evidence, Qualitative researcher experience, Trials

## Abstract

Researchers working in the field, the places where research-relevant activity happens, are essential to recruitment and data collection in randomised controlled trials (RCTs). This study aimed to understand the nature of this often invisible work. Data were generated through an RCT of a pharmacist-led medication management service for older people in care homes. The study was conducted over three years and employed seven Research Associates (RA) working in Scotland, Northern Ireland, and England. Weekly research team meetings and Programme Management Group meetings naturally generated 129 sets of minutes. This documentary data was supplemented with two end-of-study RA debriefing meetings. Data were coded to sort the work being done in the field, then deductively explored through the lens of Normalization Process Theory to enable a greater understanding of the depth, breadth and complexity of work carried out by these trial delivery RAs. Results indicate RAs helped stakeholders and participants make sense of the research, they built relationships with participants to support retention, operationalised complex data collection procedures and reflected on their own work contexts to reach agreement on changes to trial procedures. The debrief discussions enabled RAs to explore and reflect on experiences from the field which had affected their day-to-day work. The learning from the challenges faced in facilitating care home research may be useful to inform future research team preparation for complex interventions. Scrutinising these data sources through the lens of NPT enabled us to identify RAs as linchpins in the successful conduct of a complex RCT study.

## Highlights

• Successful implementation of RCTS depends on the tenacity, commitment, and thoroughness of researchers.
• Researchers with local knowledge can adapt procedures to fit the locality, supporting recruitment and engagement.
• Normalization Process Theory provides a theoretical lens to examine the work of researchers in RCTs.
• Researcher team meetings enable opportunities to share challenges, reflecting on research to build knowledge.
• Team meetings and debrief discussions, when documented, provide rich data sources of researchers work

## 1 Introduction

New patient care practices are usually implemented and tested through Randomised Controlled Trials (RCT) meaning patients in the intervention arm receive the new care practice and those in the control arm do not. However, care delivery does not happen in experimental conditions rather an interplay of social norms and practices influence how clinicians’ deliver the intervention and how patients receive the new care practice. A process evaluation study may run alongside an RCT to explore how contextual factors affect the implementation and outcomes of new care regimes (Skivington et al., 2021). The methods used in process evaluations are usually grounded in implementation theories. A theory often used in healthcare interventions is Normalization Process Theory (NPT) (Finch et al., 2012; Huddlestone et al., 2020). This mid-range social theory focuses on the work that stakeholders undertaken to embed and normalise new care practices into day-to-day practice (May & Finch, 2009). In this paper we report on the often-missing element in understanding how RCTs work, namely the activity of the trial delivery researcher. By examining research team meeting minutes and debriefing discussions in an intervention study set in older people care homes in the United Kingdom (UK), we aim to delineate and make visible everyday relational activities that operationalise a research project. First, we outline the role of researchers in complex intervention studies, then provide context on the complexities of conducting research in care homes, followed by a description of the trial. We examine documentary evidence through the lens of Normalization Process Theory (NPT) with the aim of describing and demonstrating the work undertaken by the RAs that supported effective trial implementation.

## 2 Background

Complex multi-site randomised control trials (RCT) often depend on locally based researchers to undertake recruitment and data collection at each site. This day-to-day field research is generally undertaken by research assistants or research associates (RAs) contracted to the study. They are unlikely to have been part of the research development team, yet they need detailed knowledge of how the research will be implemented. While RAs are frequently trained in the trial process operating procedure and data collection protocols, the ‘ethics in practice’ are rarely attended to (Aluwihare-Samaranayake, 2012). Yet the RA has responsibility for transparent recruitment, informed consent and robust data collection so the quality of the trial rests with the field (KIngori, 2013). RAs need to problem solve in the moment and adapt processes to fit local contexts within the constraints of approved protocols. At project initiation, local challenges may be unknown. ‘On the job’ ethical and procedural decisions and actions are often not formally acknowledged. Yet RAs are continuously making complex analytical decisions around capacity to give consent (Hubbard et al., 2003) and data management. The process and outcomes of such decisions sustain research working relationships and personalise the study, for participants and stakeholders. The skills, experience and continuing dynamic relational activity through which RAs construct and embed the research, are typically unrecognised and undervalued. Yet these skills mean that participants are retained, and rigorous data collection completed; such outcomes are vital for delivery of a successful RCT.

Since the mid-1990s, there has been increased interest in addressing the complexities of researching in care homes. Recruitment to RCTs can be time consuming and resource heavy (Ellwood et al., 2018; Reed et al., 2004; Ruckdeschel & Van Haitsma, 1997; Shepherd et al., 2015). A systematic review by Lam and colleagues (2018) identified specific challenges and barriers to conducting clinical, observational, survey and epidemiological research in long-term care facilities. Challenges included: recruiting care homes and residents, gaining consent from residents with and without capacity, retaining research staff, and methodological and budgetary constraints. An international comparative study found care home staff were interested in research, but research was perceived as “*too much to deal with*” unless there was clear management support for staff to be included effectively (Gina-Garriga et al. 2020). It was identified that care home staff wanted research to be “*on the residents’ terms*”, to be of benefit to residents and to be included in organisational everyday routines (Gina-Garriga et al. 2020). Care home staff may be concerned that research involves scrutiny and inspection of care home procedures (Lawrence and Banerjee, 2010). While recruiting to a qualitative study examining symptom control, treatment strategies and communication with families, Gonella (2021) reported that some care home staff “*were suspicious of their research project and were not interested in being part of the study*” because they considered the researcher in a position to judge their care work (Gonella et al. 2021, p. 183). These are identified challenges that the RA working in the field must manage successfully to ensure research, designed to optimise care of vulnerable adults with complex needs, is conducted to the highest standards.

RAs are known to address the challenges of research in care homes in a number of ways. An evaluation of research-ready care homes found there was engagement in research processes when there were pre-existing relationships established with research teams and the RA had knowledge of the care home's organisation cultures (Davies et al. 2014). Positive working relationships with care homes may be developed by increasing transparency of research aims and adopting collaborative approaches to implementing new ways of working (Lawrence and Banerjee, 2010); realistic timescale planning for the study (Lam et al. 2018) and ensuring sufficient time and privacy to carry out interviews with older people (Hall et al., 2009). When care home staff have been supported to understand specific research aims and data collection focus, staff collaboration and engagement with research has improved (Lawrence and Banerjee, 2010).

## 3 Study context and researcher's roles in the CHIPPs study RCT

To provide context to the data collection and RAs’ activity reported in this paper we briefly summarise the RCT design and the planned work of RAs working in the field i.e. undertaking recruitment and data collection rather than the office-based trial management research team. The trial was a pharmacist-led medicines management intervention with a particular focus on reducing drug burden and risks of falls in older people living in care homes: The Care Homes Independent Pharmacist Prescribing Study (CHIPPS). The RCT was delivered over 4 phases (internal pilot study during 2018, then 3 phases between 2018 and 2020). The RCT process evaluation is reported elsewhere (Birt et al., 2021).

**Fig. 1:**
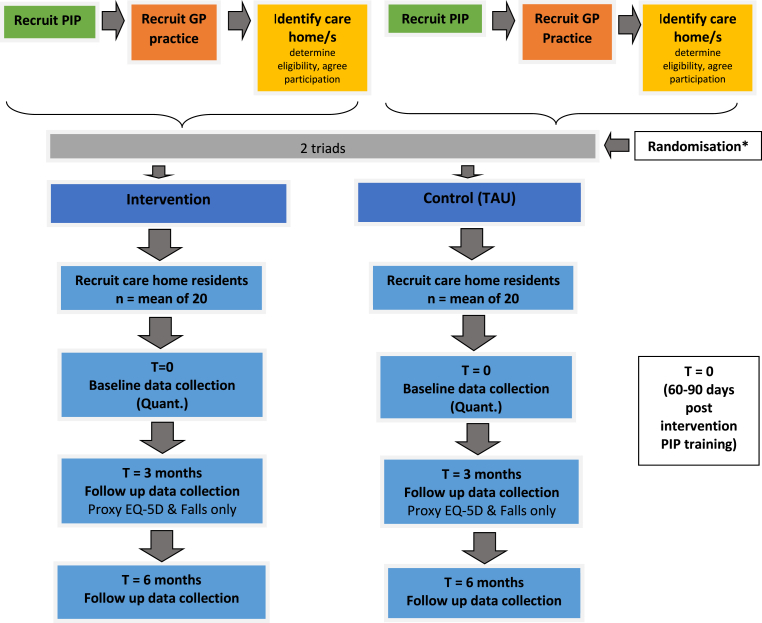
Researcher activities flow chart per intervention and control triad ∗Randomisation managed at UEA [administrative lead university], local Researchers involved in care home resident recruitment remained blind to allocation.

The trial was carried out in four geographical areas of the UK (Scotland, Northern Ireland, Yorkshire and Norfolk), and co-located with each collaborating University. RAs were employed by each collaborating university. There were four Principal investigators (PI) one in each location. PI were co-applicant on the research and had leadership responsibility for the study. Involved in the weekly meetings along with the RA were a clinical trials unit (CTU) manager and a project co-ordinator. RAs managed recruitment and data collection in parallel see Fig. 1. Once the RA had recruited a triad of General Practitioner (GP), pharmacist independent prescriber (PIP) and care home(s) they supported each triad to recruit a maximum of 24 care home residents. During the 30-month study RAs recruited 49 triads and 882 residents (with and without capacity to consent) from 72 care homes.

### 3.1 Researcher training

Prior to the RCT starting, each RA received in-person training in taking consent and data collection methods. Those joining during the trial received training from colleagues already in post. See Box 1 for training activities.

Box 1 **Research Associate Training**

RA Training covered:

• Resident recruitment and consent processes: face-to-face, via personal consultee or nominated consultee (or Welfare Power of Attorney in Scotland).
• Capacity assessments for ‘in person’ resident participant consents.
• Resident changes in capacity.
• Triad participant and resident withdrawal procedures.
• Paper and electronic Case Report Form (CRF) recording and completion.
• Completion of in-person and proxy completed validated measures (EQ-5D-5L and Barthel Activities of Daily Living Index (ADL))
• Data entry training for REDCap the information management system used by the clinical trials unit
• Safety management monitoring plan
• Researcher blinding procedures.

Alt-text: Box 1

### 3.2 RAs’ work on the study

RAs had responsibility for recruitment and data collection in their own locality. There were differing ethical and procedural considerations across locations. For example, in Northern Ireland it is usual for residents to keep their own GP so multiple care homes needed to be recruited to recruit approximately 24 residents managed by one GP practice. The study protocol applied to all four sites despite local differences in how GPs were allocated to care home residents.

Recruitment was complex because independent individual consent was required: first from the pharmacist, GP and care home manager(s) within each triad and then the resident. At the point of recruitment, the RA communicated roles and responsibilities to the pharmacist and GP to optimise subsequent fidelity to intervention delivery. GPs then identified and sent invitation packs (letter of invitation, participant information sheet) to care homes and residents to participate to complete triad recruitment.

Consent of older people in care homes needs careful management and took significant time. The RA first arranged a face-to-face meeting with the resident to confirm capacity to consent to participation using a standard approach (Hooper et al., 2016). They then provided a verbal overview of participant information. Signed informed consent was obtained or declined at that point. For residents without capacity, the researchers provided invitation packs to the consultee (often the care home staff were consultee). In Scotland, if next of kin were not available extra time was needed to send information to the Welfare Power of Attorney, as care home staff cannot be consultees.

Once the study had started RAs visited each care home and GP surgery to undertake data collection. Each RCT phase had three data collection points during the 6-month delivery period in both control and intervention arms, see Table 1.

**Table 1:** Data collection time point, data source and data collected by researcher in each study phase.

Data Collection Point	Data source and data collected
Baseline	GP records: data on resident participant's previous and current health problems; prescriptions for chronic and acute conditions or the last 3 months; medical tests, investigations, hospital admissions and other health service contacts for the last three months
	Care home records: data on health and social care contacts and descriptions of falls for the last 3 months; care home status (residential or nursing care.)
	Resident completed tools; researchers supported resident participants with capacity to complete the EQ-5D-5L face to face measure.
	Care home staff completed tools: the Activities of Daily Living (ADL) (Roper et al., 2000) for all residents and the proxy version of the EQ-5D-5L for resident participants without capacity.
3 months (midpoint)	Care home records: researchers extracted data on falls and adverse drug events occurring in the last 3 months; any changes to care home status,
	EQ-5D-5L: completed by residents or by care home staff and researcher as above.
6 months (end of intervention)	GP records: repeat of baseline data collection
	Care homes records: repeat of baseline data collection (falls, hospital admissions and adverse drug events in the last 3 months
	Resident completed tools: researchers supported resident participants with capacity to complete the EQ-5D-5L face to face measure.
	Care home staff completed tools: ADL (for all resident participants) and EQ-5D-5L proxy measures for residents without capacity

Table 1 Data collection time point, data source and data collected by researcher in each study phase.

## 4 Methods

The process evaluation for the RCT had ethical approval to examine study documents: English ethical approval was gained from East of England Cambridge Central Research Ethics Committee 17/EE/0360 (28.11.2017). Scottish ethical approval was gained from Scotland A research Ethics Committee 17/SS/0118 (07.12.2017). Authors attended research team meetings during the RCT and contributed to end-of-study RA debriefing discussions. The secondary analysis reported in this paper developed from our awareness of the central position of RAs’ roles and contributions which were reinforced during the process evaluation. The new understandings generated are positioned within a social construction paradigm, asserting that reality and meaning are subjective and develop through social processes and interactions within social groups (Crotty, 1998).

## 5 Data sources

One data source were data are 129 sets of meeting minutes: 89 sets were informal minutes generated from weekly research team meetings from February 2017 to April 2020 (present were RAs, CTU manager and project co-ordinator; 40 sets of formal minutes from monthly Programme Management Group (PMG) meetings (present were all PIs, RAs, CTU manager, study co-ordinator, and wider team such as statistician, health economist, NHR trust representative and public and patient involvement colleagues). These research records covered study set up, recruitment and data collection through all phases of the RCT. The second data source came from the two debriefing group meetings held with seven RAs at the end study.

### 5.1 Research team meetings

Meetings took place virtually via teleconferencing services and were facilitated by CTU manager and the study coordinator. All researchers were expected to attend and contribute. The meeting purpose was to monitor and support RAs to meet recruitment targets and monitor adherence to data collection, to provide guidance and support problem-solving where necessary. The meeting notes were reviewed for accuracy, shared with PIs and filed in the RCT site folder.

### 5.2 Programme Management Group meetings

During intensive periods of data collection, RA meetings were suspended. Instead, PMG meetings were attended weekly by RAs. Here recruitment and data collection were discussed. A pragmatic decision was taken to include PMG minutes in the data set minutes to ensure continuity.

### 5.3 Researcher debriefing discussions

All researchers were invited to two end-of-study debriefing discussions facilitated by a qualitative researcher (LB) as part of peer validation of the trial process evaluation. Detailed notes were taken to record discussion points, consensus, and variance between seven researchers across the four localities.

## 6 Data management and analysis

Within the trial process evaluation, two qualitative researchers (LD and LB) collated 129 sets of meeting minutes (40 ​PMG and 89 weekly researcher meetings). A random selection of 20% of minutes were used to develop an inductive coding framework to organise and categorise the work RAs were doing (Willis et al., 2012). The coding framework was started by (LD) and reviewed and discussed with (LB). The framework categorised codes into barriers and facilitates and sorted data into the stages of implementation the study protocol and recruitment to the study. Sub-codes in each category were: communication channels, data collection tasks, environmental factors and factors relating to the Pharmacist Independent Prescriber as they were the key person undertaking the intervention. NVivo software supported data organisation. Consensus was reached and all meeting minutes were coded. During coding, to assist refinement and clarification, regular meetings took place between the two researchers, the RAs, study-coordinator and PIs. The indicative themes developed were peer validated during the two end-of-study researcher debriefing discussions.

During this inductive data analysis, we noted that much of the work and decision-making reported by RAs was essential to the implementation and normalization of the intervention. We were using NPT to understand what had happened in the RCT so had an increased awareness of the components. Therefore, we deductively reorganised the data to examine it using the lens of Normalization Process Theory (NPT) to observe and consider how researchers normalised their own work. NPT is a sociological middle-range theory which supports critical consideration of processes through which people perceive an intervention, implement it, and develop new relationships and working practices (May & Finch, 2009; May et al., 2009).

NPT organises data into four conceptual constructs: coherence, cognitive participation, collective action and reflexive monitoring. Coherence refers to sense-making work [differentiation; communal specification; individual specification and internalization] needed to share relevant ideas of the work. Cognitive participation refers to relational work [initiation; enrolment; legitimation and activation] needed to mutually orientate actions; Collective Action refers to operational work together [interactional workability; relational integration; skill set workability and contextual integration]. Reflexive Monitoring refers to appraisal work [systemisation; communal appraisal; individual appraisal and reconfiguration] through which actors can review the effects of their actions and if necessary, adjust ideas and relational roles.

We report documentary evidence from trial minutes to support interpretations. To ensure the research team's confidentiality identifying initials are removed and roles defined, location is recorded by city of the collaborating university.

## 7 Results

**Fig. 2:**
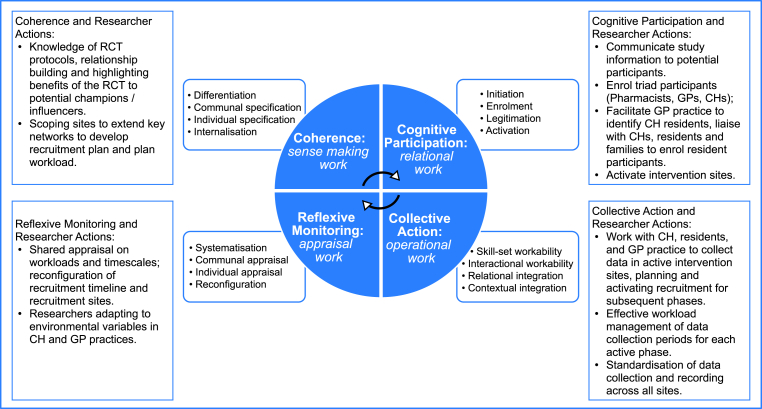
Normalization process theory core constructs and components.

The results situate the work of RAs and relates this activity to the four constructs of NPT: Coherence – sense-making work; Cognitive participation - relational work; Collective Action - operational work; Reflexive Monitoring - appraisal work (see Fig. 2).

Fig. 2 Normalization Process Theory Core Constructs and Components.

### 7.1 Coherence: sense making work

Examining the RAs’ work, as they worked within study protocols to implement and collect data for a community-based trial, through the NPT construct of coherence made apparent the sense-making work RAs did for themselves and others as they came to understand and adapt with new systems. There was evidence of RAs striving to quickly build effective relationships with all stakeholders (including GPs, pharmacists, care home residents, care home managers and staff, and relatives) to ensure recruitment met study targets and to build a shared understanding of the benefits of joining the trial.

RAs worked independently in their locality to understand working relationships between GPs, care homes and pharmacists, having regular contact with each to explain the distinct activity each would need to do to bring about a process change. They developed and drew on their local knowledge of governance and care systems to access potential triad participants. Team minutes illustrated the scoping activity that supported recruitment:

Extract 1 Research Team Meeting Minutes: (21.02.2017)

ALL field RAs for all phase planning “RAs to find out how many pharmacists in area for this year and next year if possible.”

ABERDEEN 25.4.2017 “site PI (Principal Investigator) has arranged for local RAs to present the [study name] at the Health Board Lead Primary Care Pharmacist meeting on 11th May. This will hopefully help recruit pharmacists for WP6…PI and 2 RAs to attend RA1 to do power point presentation”.

Here we see work being directed and supported by others but as understanding and confidence develops the RAs take the lead in decisions.

Eighteen months into the study the RAs are making suggestions and have the knowledge of the research field to lead on how best to implement recruitment:

Extract 2 Research Team Meeting Minutes: (23.10.2018)

Phase 1 “RA and PI have slightly amended the expression of interest letter to go out to GPs as they will be approached through the CRN (Clinical Research Network) - the wording now reflects that GPs with their own pharmacist are being sought for the study.”

This modification to study documents illustrates ways RAs recognised and addressed different primary care structures in each location, highlighting the importance of them being able to draw on local awareness to recruit.

In the weekly meetings RAs were enabled by a trial manager to explore and share pragmatic solutions to recruitment through discussing and documenting experiences of recruiting triads and care home residents. When deviations from protocol were noted, they negotiated and agreed how to proceed:

Extract 3 Research Team r meeting Minutes (12.03.2019)

ABERDEEN Phase 2: “WPoA (Welfare Power of Attorney) consent forms with ticks in boxes (no initials). Trial Manager confirmed that these forms can be accepted – need to ask the signatory once to initial. If no response, no need to chase further but [RAs] must do a file note.”

In this multi country study minutes further illustrated the importance of local knowledge during consent processes. In Scotland RAs had to be aware of legal differences and build in additional processes and time to adhere to legislation about recruiting residents without capacity.

In this study all RAs had previous experience of research in the locality and the study design provided them with time and opportunity to find and share local knowledge. This supported recruitment of multiple sites within each locality.

### 7.2 Cognitive participation: relational work

During recruitment of a triad, RAs needed to organise relational work to build an efficient working community of practice across multiple sites exemplifying cognitive participation. This meant they needed to quickly build strong relationships with all stakeholders: GP, Pharmacist, care home staff. This was particularly important in the control arm where only data collection happened, removing the opportunity for more developed social connections. RAs needed be able to communicate with different groups of people to share different types of information. RAs maintained close contact with GPs, often by telephone and through practice managers, to share bounded pragmatic information on when tasks had to happen so RAs could meet their target of recruiting care homes and residents within trial timelines. Within care homes key contacts were managers, deputy managers and senior nursing staff and here RAs had to adapt communication to share complex clinical information about what would be required of care homes during the trial and ascertain if they were willing to adopt new ways of working. RAs needed to plan their own time so the sequencing of invitations across the triad was efficiently managed:

Extract 4 Research Team Meeting Minutes (03,01.2018)

ABERDEEN: Pilot Phase “GP practices will send out formal invitation out to Care Homes this week and RAs will visit Care Homes next week to gain formal consent.”

RAs needed to be adaptable and able to respond to changes in trial sites. Here a change in personal threatened to derail recruitment. A suitable alternative contact was identified but the research team recognised delay may be inevitable:

Extract 5 Research Team Meeting Minutes (09.01.2018)

BELFAST Pilot Phase: “Visiting the care home on 10/1/18 to carry out Site Initiation Visit (SIV). The Belfast triad has 3 care homes and the manager for one of these is on annual leave for the next 2 weeks so unable to carry out SIV [Site initiation visits] until 24th January (written consent already received). Project co-ordinator suggested seeing if a deputy can sign the agreement, however RA may need to wait.”

RAs need to be adaptable as recruitment did not occur in a consistent way. At some sites, pharmacists were recruited first and approached their GP surgery to join the study. At other sites, interested GP surgeries approached their practice pharmacists. Interested GPs either discussed the study with care home managers informally during planned visits, or invited them to attend formal meetings with RAs, often with Practice Managers present. Where direct contact with GPs was not possible RAs worked more closely with pharmacists and Practice Managers, who were then influential in recruiting a GP. However, minutes clearly indicate distinct roles for each stakeholder and the rationale for their role:

Extract 6 Research Team Meeting Minutes (16.01.2018)

Any Other Business “RA queried when lists of patients could be generated by GP practice.”

Trials manager responds: “Intervention scheduled to begin 26 March. Patient lists to be generated six weeks beforehand. Exact week beginning date to follow. Need balance of having sufficient time to recruit but not so much time that participants are lost after consent but before the intervention. RAs reminded to liaise with GP practice to generate list of patients. This is not a task for the pharmacist.”

RAs were required to balance the trial deadlines alongside navigating GPs’ busy schedules and competing demands – maintaining professionalism throughout. It was important for GPs to understand the inclusion and exclusion criteria of care homes and residents to ensure only eligible care homes were approached, as at times RAs contacted the home manager and found the home did not have sufficient residents to meet the inclusion criteria. The work they did to progress recruitment was also disrupted when Care Quality Commission inspections placed a care home into special measures and the manager decided to withdraw from the research.

### 7.3 Collective action: operational work

Collective action is the NPT construct that addresses operational work needed to implement a new practice or ‘way of working’. The operational work of the RA here entailed collecting study data across four phases and supporting GPs, pharmacist and care home staff to undertake the work needed for the intervention to be implemented in the workplace. RAs had to manage overlapping tasks, working in the high-pressured care home environment and adapting to challenging data collection methods.

During the internal pilot phase and Phase 1, minutes revealed RAs had difficulty simultaneously managing numerous different and competing trial tasks. For example, internal pilot phase data collection coincided with recruitment for Phase 2 (See Fig. 1). The following examples from minutes demonstrate in more detail how intensity of workloads required RAs to adapt their working practices:

Extract 7 Research Team Meeting Minutes (30.04.2019)

ABERDEEN: “Phase 1 – is booking in 6 months visits to collect data.

Phase 2 – GP baseline data collection hasn’t started yet for phase 2. All care home Baseline data is collected

Phase 3 – Site xxx is looking promising with 3–4 triads potential. RA has liaised with them and will arrange to meet with the GPs soon.”

Extract 7 illustrates how trial tasks overlap. Extract 8 demonstrates how the RA manages overlapping workloads:

Extract 8 Programme Management Meeting Minutes (02.07.2019)

BELFAST: “PI confirmed that [Ph 3] recruitment was underway but has so far been slow, with only 5 participants recruited so far. Reminder letters have been sent and care homes have agreed to stand in as nominated consultees where necessary.

There was concern expressed that triad B1 could only recruit a maximum of 7 residents, and there was some discussion about how this shortfall could be made up. It was stressed that in every location the aim should be for every triad to have 24 participants recruited if at all possible.’

Situational factors impacted on care home data collection visits, specifically the high-pressured working environment of a care home. RAs all stated care homes were affected by chronic low staffing levels and management changes, sometimes affecting care home staff participants' awareness of the study and/or the purpose of researcher visits. In this operational work diplomacy and professionalism was required from the RA. For example, when a pharmacist unexpectedly withdrew from the intervention, the care home manager expressed disappointment about having to discontinue the study especially with the effort expended to recruit residents and involve families. Drawing on strong relationships established with the care home, the RA was able to manage the disappointment successfully and secure access to the care home for final data collection. Situational factors often involved RAs ‘thinking on their feet’, adapting quickly to their environment and responding within the bounds of ethics and trial protocol.

While RAs' relationships with care home staff were usually positive the debriefing discussion highlighted, they faced challenges in data collection. Care home managers understood the purpose of data collection visits but sometimes RAs were told that these visits added to care home manager's workload. While most care homes were receptive to RAs entering and collecting data, one RA reported having to work in a space ‘the size of a cupboard’. This space was a dedicated ‘admin space’ within the care home where care plans were kept, and a better space could not be offered. Others reported care home staff left them to trawl residents' notes for data on falls within public spaces of the care home, raising concerns about confidentiality. In public spaces RAs stated they were often mistaken as care staff by residents and were summoned to help or assist. RAs experienced ethical concerns when residents were trying to get up from a chair unassisted and felt uneasy ‘just watching’. In such instances, the RA alerted care staff to the situation*.* These types of role conflicts were not reported in the more formal weekly meetings but were recognised during the debriefing meetings as together RAs acknowledged how they navigated such stressor within a typical care home data collection event, with all saying they often left the care home feeling exhausted.

Methods of data collection needed to be ‘re-operationalised’. All RAs attending the debriefing meetings reported on resident participant challenges that arose in care homes on the day of visits meaning they need to adapt ‘on-the job’. For example, hospitalisation or death of a resident participant; loss of resident's capacity to consent to completion of self-reported measure required subsequent amendments to data collection. RAs who received data directly from care home managers, rather than carrying out self-searches, reported data collection was disrupted by numerous *ad-hoc* care issues that required the manager's immediate responses. These situational factors impacted on time taken to complete data collection tasks. RAs reported needing to respond sensitively, patiently, and flexibly to all situations that arose during care home visits.

Standardised approaches to data collection are necessary to ensure data are recorded within pre-specified trial protocols. For example, RAs discussed procedures for recording falls during research team meetings. Debrief meetings confirmed the same data was being collected in relation to residents’ falls however data was collected in different ways in different locations. Some RAs were verbally given the falls data by the care home manager whereas others were given care home records and had to self-search for relevant data. This variation meant RAs needed to deploy different examining and recording skills. For instance, those who received data verbally needed to use good verbal communication skills to check detail and confirm verbatim recording. When RAs were provided with electronic and paper data sources on falls, they extracted the data as verbatim extracts from resident and care home records. The RA then checked what they had recorded by returning a second time to the records. These steps were taken to confirm data accuracy and relevance to the trial outcome measures. This demonstrated relational integration work and the need for collective action. Minutes indicated RAs new to the research field did not have the same level of understanding of data collection approaches as those who were experienced in the field:

Extract 9 Research Team Meeting Minutes (30.09.2019)

LEEDS “Re [triad] L27 The falls data collected from L27 has been found to have errors, when checking [temporary staff name] collected falls [data] against the care home records, RA found 30+ additional falls

Discussion has happened between with [temporary staff] and [RA]. [RA] has explained it was consequence of a computer system error

The [RA] also found other errors at other care home

[RA] has already 4 GP visits booked in, therefore has asked all care homes to gather all the falls data from the 4th December. She will then go through the data again and check through for errors.

Trial Manager suggested to have a file note made on this issue – detailing the ‘error’ and the measures [RA] takes to correct.”

The research protocol formally set out RA activities, but individuals were able to reflect on and adapt systems of working to increase efficacy. For example, in modifying processes to streamline data entry:

Extract 10 Research Team Meeting Minutes (12.02.2019)

“Trial Manager has developed a new process to allow users to directly upload participant information. This is being piloted by RA currently. She will feedback to the group.”

Within GP practices data collection had specific challenges that related to ‘Contextual Integration’ roles as each surgery operated differently. To access relevant data sources at each site, RAs had to be prepared to inform surgery staff of their role and purpose of visit, to identify Health Board and IT contacts, and to adapt quickly to workspace allocated whether hot-desking in busy reception areas or in remote offices. During debriefing meetings RAs reported how securing health board and individual surgery passwords was sometimes difficult because health board/surgery IT staff had competing demands on their time or worked part-time. Also, organising access to a free desk space and computer took time. Once passwords and desk space were secured RAs had to find ways to understand functions of the different software systems used to report and extract data. These negotiations and new learning made fieldwork and data entry more complicated to organise and slow to complete; this coupled with limited time allocated to a desk led to multiple return trips to GP practices.

The third phase of data collection was affected by the first national COVID-19 lockdown: none COVID-19 research was nationally suspended and working practices changed across universities and GP practices. This led to data collection in two GP practices being delayed by 3 ​months ​as protocols on socially distanced in-person access were negotiated between universities and GP practices.

### 7.4 Reflexive monitoring: appraisal work

Reflexive monitoring is the appraisal work undertaken to see how embedding processes and practices into real work situations happened: what worked and what did not work for the research team and participants. RAs continually undertook reflexive work though each phase of the trial. This enabled learning and normalization of practices to be carried forward. There were three key areas where RAs reflected and adapted practice: when residents had fluctuating capacity to give consent, when data collection procedures needed to be adapted due to COVID-19 infection control procedures and when, in phase three, recruitment needed to be maximised to ensure sufficient statistical power for analysis.

An example of reflexive monitoring lay in understanding challenges posed by care home residents not having capacity to give consent and the legislative differences across geographical areas. The additional time taken to recruit resident participants because of differing contextual requirements had not been factored into the study design. Therefore, recruitment times were considerably extended, leading to delayed starts in each phase, complicating administrative processes. When RAs appraised processes of gaining consent, they stated that they would have benefited from detailed training on their responsibilities under the Mental Health Act or other UK national legislation. This would have enabled them to be able to make judgements when decisions were unclear:

Extract 11 Research Team Meeting Minutes (06.11.2018)

BELFAST Phase One: “RA queried the position regarding a resident who was unable to communicate any responses, but the care home had identified as having capacity. It was clarified that in this case [researcher] should detail the interaction on the Capacity form and go ahead with contacting next of kin.”

In Phase 3 when planned resident recruitment targets were not reached, reducing the a priori statistical power in the primary outcome. RAs and PIs had discussions that led to a decision to increase the number of triads by extending activities beyond original geographic boundaries. This pragmatic decision increased workloads for RAs in Scotland where seven triads were recruited instead of the original four:

Extract 12 Research Team meeting Minutes (12.03.2019)

ABERDEEN Phase 3: “RA is still hopeful about recruiting 4 pharmacists, though it may be necessary to recruit from other regions (Highland/Fife) and so bearing in mind additional paperwork/ethics considerations etc, the start date was likely to be deferred.”

Extract 16 Programme Management Team meeting (13.08.2019) Minutes:

“Belfast, Leeds and Norfolk have completed Phase 3 recruitment now and the intervention has started for all triads in these sites other than two in Leeds who would be starting shortly. In Aberdeen, four of the seven triads will be commencing the intervention on Monday 19 August.

Recruitment is ongoing in all triads in Aberdeen and PI advised the group that so far 70 participants had consented for Phase 3.”

RAs had set protocols to follow to support fidelity in data collection, however team minutes indicate variance in the activity due to public health restrictions inherent in COVID-19 pandemic. This extract evidenced RAs ability to appraise and reconfigure work:

Extract 13 Research Team Meeting Minutes (24.03.2020)

ABERDEEN Phase 3: “Trial Manager agreed RA should suggest collecting all data by phone but if this was not possible, to at least attempt to collect all falls data.”

However not all sites were receptive to immediate changes in procedures, which threatened integrity of the trial:

Extract 14 Research Team Meeting Minutes (24.03.2020)

ABERDEEN “RA has emailed the GPs regarding the last two Phase 3 triads. One has said no to any data collection at this time. The other has agreed but not yet.

RA has emailed both care homes for the last two triads but has had no reply. Will try them again in a couple of weeks when things may have settled down, and due to ‘having no visitors’ they may have a little capacity to consider our data collection.”

The reluctance of intervention providers to allow RAs access to data during the early stage of COVID pandemic meant that data collection was delayed while in this study RAS had contracts extended to enable them to recommence data collection when new risk assessments were in place, without this trial data would have been lost.

RAs initiated reflexive monitoring which helped the wider research team such as statistician and process evaluation lead anticipate, and then adjust workload changes in changing circumstances.

## 8 Discussion

RAs working in the field undertake essential work to embed the key components of trial protocols into their everyday working. Using NPT to examine accounts of RAs within a RCT highlights the active work RAs do to make sense of an intervention, to build relationships to enable others and themselves to embed new working practice and the skills they need to reflect on what is working and what needs changing to progress the trial. This brings to the fore the RA's role in supporting successful delivery of RCTs and the complexities that can occur when research is delivered across national sites, with health professionals from differing disciplines. Our results are likely to have relevance to others planning and undertaking research within care homes, and other care settings where there are distinct challenges in research involvement. We now situate our findings within the wider literature on care home research and implementation science. We critique whether NPT may be an appropriate framework through which to examine and understand researcher and stakeholder ‘buy-in’ to research processes.

### 8.1 Responding to challenges of care home research

Many aspects of care home research are recognised as challenging, particularly when recruiting to complex RCTs (Ballard et al. 2018; Froggatt et al. 2016; Jenkins et al. 2016; Lam et al. 2018; Zermansky et al. 2007). Yet we found when RAs developed a strong sense of the meaning of the intervention, they could effectively communicate intervention requirements to differing health professionals and care home resident participants. The complexity of working directly in care homes, and with residents with and without capacity resonates with findings reported by Lam et al. (2018), Ballard et al. (2018) and Shepherd et al. (2015) on how this requires specific researcher interpersonal skills, knowledge and full commitment to the trial processes (Lam et al. 2018). More recently, Fossey et al. (2020) considered the potential for maximising care home staff engagement in future complex RCT research by researchers working more collaboratively with care homes. Their study also recognised the importance of positive relationship building with care home staff and the value staff contributions bring to research. Collaborating directly with care homes enabled any unanticipated consequences of research work being better understood by care home staff and researchers (Fossey et al. 2020).

Our finding that changes in care home management or residents’ wellbeing necessitated the RA to replan their work resonates with Lam (2018) who noted that high staff turnover in care homes, care home attrition and resident turnover impacted negatively on home and participant retention rates and study timescales. We found a named RA liaising with care homes in each area was essential to maintain relationships; evidenced by retention of consented care homes. This resonates with Lam (2018) who proposed identifying a key researcher to develop long-standing relationships with care homes during research design and planning as this could minimise any negative consequences from being involved in research experienced by care home staff.

#### 8.1.1 The researcher as a social instrument

NPT proposes that people are the vectors of implementation and throughout this study we see the active daily work of RAs changing and adapting pre-determined ways of working to meet local need and ensure the recruitment and implementation of the intervention happened. The seminal work of Timmerman and Berg (1997, 2003) proposes that universality through standardised protocols and procedures is always buffering against locality where past, present and potential future networks and practices shape what people do when implementing new practices. They also suggest that it is the outcome not the protocol which is important to those who receive or clinically deliver the care. In research the RA is the person presumed to ensure universality through implementing research protocols. However our data illustrates this as RAs drew on their personal local knowledge and networks to support recruitment. RAs experience in care home research and pharmacy-based interventions differed and this led to different approaches to data collection affecting accuracy across researchers. Although patient care was not directly impacted here Lawton et al., 2012 report how personal and professional beliefs and motivations may directly affect trial implement especially when researchers do not have ‘buy into’ the intervention.

The challenge in being in situ for data collection was evident in RAs accounts of how much involvement they should have with residents in the public spaces of the care home. Although they were not undertaking ethnographic work RAs need to demonstrate understanding of GP and care home working practices to gain access and make requests in appropriate ways. There were accounts of role shift such as whether to intervene to prevent a resident falling (Adler and Adler, 1987). The urgency of the situation means that the opportunity to remain non-participant by passing on requests for help may not be always possible in the dynamic care home (Cross et al., 2022) When RAs encountered such challenges during fieldwork the weekly research team meetings provided a safe forum for considering and resolving arising issues. In this study RAs did not report that quality of data collection was affected but did say concentration was harder and they left the care home exhausted. It seems relevant to explore further whether complex numerical data collection such as number of falls or clinical episodes may be affected by the places this happens. This point resonates with work of others who identified the importance of building-in more flexibility to research budgets, research staff support and training, and timeframes to help researchers adapt to unanticipated situational challenges commonly encountered when working with care homes (Hall et al. 2009; Lam et al. 2018). While it may not be usual to have weekly research team meetings within trials, the experiences of RAs in our study suggest it is a helpful resource. The low turnover of RAs during the study may have indicated the value they, and potentially others within the research team, placed on their role (Hudson, 2021). Retention of RAs may be important as they have a crucial role in maintaining relationships with participants and effective collaborative relationships with participants.

### 8.2 Using NPT as a framework to explore the researcher's work

We found that without consciously realising it RAs were undertaking the work actively theorised in NPT. The application of this mid-range social theory was appropriate as it helped us understand the local contexts within which RAs were working and so to explore ways they worked individually and collectively to implement the protocol (Murray et al., 2010) The application of NPT specifically on RA activities enabled us to better understand the complex nature of their roles (McNaughton et al., 2020).

Importantly if RAs can support understanding and commitment (coherence) to collective action in practice with care home managers and staff early on, this might pre-empt research related challenges such as care home staff seeing intervention RAs as ‘interfering with’ or ‘spying on’ existing working practices (Lawrence and Banerjee 2009: 420). Care home staff can experience feelings of being additionally burdened by research data collection in care home settings (Ballard et al. 2020). There may well be extra burden placed on care home staff if, as in this trial, they are expected to spend time collecting data for residents with complex health conditions. Early preparation and clarity of what the trial will entail may help stakeholders operationalise intervention procedures.

The capacity of RAs to dynamically develop their skill set could be seen as being central to the success of the trial as they continually needed to reflect on progress and consider what was working and why. Research team weekly meetings provided opportunities for individual and communal appraisal and reconfiguration of work as RA's balance timely recruitment with data collection across the study phases. However, there were limitations to the weekly meetings as they were chaired, structured and time limited, with monitoring of trial phase implementation being a primary focus. The RA debriefing and this analysis indicates that creating workspaces where RAs can mutually identify support, share experiences and fieldwork concerns may have beneficial outcomes for researcher retentions and trial success. RAs in our study suggested that formalising research team meetings and building in informal debrief time might have led to less stress when they needed to manage competing demands in geographically isolated sites.

### 8.3 Strengths and limitations

The flexibility and applicability of NPT enabled us to consider how the work of RAs facilitated implementation of a multisite complex intervention. However, we appreciate that using a different implementation theory may have brought to the fore differing interpretations. Analysing naturally occurring research documents (team meeting minutes and debriefing meeting notes) generated throughout the trial provided an opportunity to interpret the complex work carried out by the RA. However, the formal structure of meetings and the language documenting RA activities was somewhat restrictive, lacking the usual depth of explanation present in qualitative evaluation; this being a reality of using natural work products (Willis et al., 2012). Additional detail regarding individual and collective RA experiences may have been lost during the suspension of research team weekly meetings when time pressured targets took priority. Our two debriefing meetings provided context and socially situated meaning to formal meeting notes. Using debriefing discussions as a method to better understand barriers and limitations to trial implementation is likely to add detail to the process evaluation and/or future research design considerations. We undertook data analyses drawing on the concepts outlined in the many published NPT papers (May et al., 2018). Nonetheless the rigour of analysis would be enhanced by reference to May's publication on coding within NPT (May et al., 2022).

### 8.4 Implications and recommendations

Using NPT to analyse research team meetings and programme management meetings provided a novel way to appreciate and understand the RA's ‘practical’ work in the field and what types of training they may need to best support trials.

Our close inspection of the reflection and decision-making which happened in weekly research team meetings indicates such meetings serve a more important purpose than mere monitoring. They provide space for RAs to become reflexive in appraising how their own work enables them to support participants to normalise the intervention within their everyday working practices. This is important because stakeholders need to be able to readily adapt successful interventions for themselves after the research team leave the field: they need to be able to see ways to fit the intervention activity within their usual working practices to produce positive practice outcomes. Continued slippage in study timescales can lead to trial tasks overlapping. This increases workload stress for RAs and needs to be acknowledged and support offered if needed. We suggest including regular research group meetings in large multisite trial may enable active dialogue and reflexive monitoring between senior research staff and researchers in the field, providing an environment which could support constructive reflection and problem-resolution. Such meeting should include space for informal reflection, discussion of site-specific concerns and any personal concerns which might affect work.

RA debriefing meeting beginning, middle and end-of-project can also provide valuable insights to identify in more detail barriers and facilitators to recruitment and RCT implementation in constructing more locally engaged and effective future project timelines, staffing levels and recruitment targeting. Final debriefs should become routine in end-of project-closure processes. Debriefing discussions can provide opportunity to identify shared experience and shared learning, helping confirm understandings: sense-making (Peng et al. 2020). We found that the debrief meetings also served as an important means to reflect on research processes and on experiences which at times had been challenging and emotional before RAs left the research team.

## 9 Conclusion

Developing and implementing research in care homes is complex, and it is the researcher that is the conduit between the research field and research study management.

Using the lens of Normalization Process Theory to interrogate documentary evidence from research team meetings, the work of field researchers becomes explicit. Field researchers actively make sense of the intervention, develop relationships with participants to enable them to undertake the collective work needed to implement the intervention, alongside their own work of recruitment and data collection. Through these processes, researchers are reflexive, constantly appraising their own work and the collective research team's activities in order to adapt and refine procedures. Creating spaces which are less formal for researchers to debrief may make these processes stronger to the benefit of the researcher and the research.

## Funding

This is a summary of independent research funded by the 10.13039/501100000272National Institute for Health Research (NIHR) under its Programme Grants for Applied Research Programme (Grant Reference Number RP-PG-0613-20007). The views expressed are those of the authors and not necessarily those of the National Health Service, the NIHR or the Department of Health.

## Declaration of competing interest

The authors declare that they have no known competing financial interests or personal relationships that could have appeared to influence the work reported in this paper.
